# Overweight and Obesity Based on Four Reference Systems in 18,382 Paediatric Patients with Type 1 Diabetes from Germany and Austria

**DOI:** 10.1155/2015/370753

**Published:** 2015-06-01

**Authors:** M. Flechtner-Mors, K. O. Schwab, E. E. Fröhlich-Reiterer, T. M. Kapellen, T. Meissner, J. Rosenbauer, R. Stachow, R. W. Holl

**Affiliations:** ^1^Institute of Epidemiology and Medical Biometry, ZIBMT, University of Ulm, 89081 Ulm, Germany; ^2^German Center for Diabetes Research (DZD), 85764 Neuherberg, Germany; ^3^Department of Pediatrics and Adolescent Medicine, University Hospital Freiburg, 79106 Freiburg, Germany; ^4^Department of Paediatrics, Medical University of Graz, 8036 Graz, Austria; ^5^Hospital for Children and Adolescents, University of Leipzig, 04317 Leipzig, Germany; ^6^Department of General Pediatrics, Neonatology and Pediatric Cardiology, University Children's Hospital, 40225 Düsseldorf, Germany; ^7^Institute of Biometrics and Epidemiology, German Diabetes Center, Leibniz Center at University of Düsseldorf, 40225 Düsseldorf, Germany; ^8^Rehabilitation Clinic for Children and Adolescents, Westerland, 25980 Sylt, Germany

## Abstract

*Aim*. To evaluate the prevalence of overweight and obesity in paediatric type 1 diabetes (T1D) subjects, based on four commonly used reference populations. *Methods*. Using WHO, IOTF, AGA (German pediatric obesity), and KiGGS (German Health Interview and Examination Survey for Children and Adolescents) reference populations, prevalence of overweight (≥90th percentile) and obesity (≥97th percentile) and time trend between 2000 (*n* = 9,461) and 2013 (*n* = 18,382) were determined in 2–18-year-old T1D patients documented in the German/Austrian DPV database. *Results*. In 2000, the overweight prevalence was the highest according to IOTF (22.3%), followed by WHO (20.8%), AGA (15.5%), and KiGGS (9.4%). The respective rates in 2013 were IOTF (24.8%), WHO (22.9%), AGA (18.2%), and KiGGS (11.7%). Obesity prevalence in 2000 was the highest according to WHO (7.9%), followed by AGA (4.5%), IOTF (3.1%), and KiGGS (1.8%). In 2013, the respective rates were WHO (9.6%), AGA (6.2%), IOTF (4.5%), and KiGGS (2.6%). Overall, the prevalence of overweight and obesity increased from 2000 to 2006 (*p* < 0.001) but showed stabilization thereafter in girls and overweight in boys. *Conclusion*. Overweight and obesity prevalence in T1D subjects differs significantly if it is assessed by four separate reference populations. More detailed assessment of each child is required to determine obesity-related risks.

## 1. Introduction

The prevalence of overweight and obesity in childhood has increased worldwide, although it is plateauing in some countries [1, 2]. In addition, a continuous increase in the prevalence of obesity has been observed in children and adolescents with type 1 diabetes (T1D) [3, 4]. Obesity is a major risk factor for the development of cardiovascular diseases [5, 6]. T1D patients are at risk for cardiovascular diseases such as hypertension, dyslipidemia, and elevated haemoglobin A1c (HbA1c) levels [7, 8]. The presence of overweight or obesity constitutes another risk factor [9]. Furthermore, in addition to the global increase in childhood obesity, modern intensive insulin therapy (multiple daily injection therapy, continuous subcutaneous insulin infusion) is associated with weight gain in T1D patients [4, 10].

The body mass index (BMI) is widely used for the classification of overweight or obesity [11]. In children and adolescents, BMI changes markedly with age and differs between boys and girls. Thus, BMI centile curves have been developed for clinical and epidemiological purposes, specifically for the paediatric population [12, 13].

In 2006, the World Health Organization (WHO) published a growth standard for children up to five years of age based on data from healthy children around the world [14]. For school-age children and adolescents, the WHO developed growth reference charts based on the 1977 National Center for Health Statistics/WHO growth charts, supplemented with data from the WHO Child Growth Standards published in 2007 (*n* = 22,240) [13].

In 2000, the International Obesity Task Force (IOTF) used data from six large nationally representative cross-sectional growth studies (Brazil, Great Britain, Hong Kong, Netherlands, Singapore, and the United States) to develop an internationally accepted definition of child overweight and obesity (*n* = 192,727) [12].

In 2001, growth charts for German children were published based on pooled data obtained from 34,433 young subjects [15].

In 2008, anthropometric data were released from a population-based cohort of 17,641 German children from the German Health Interview and Examination Survey of Children and Adolescents (KiGGS) [16].

At present, no growth chart is generally accepted to define normal weight, overweight, and obesity in children and adolescents. Among a wide range of studies, WHO and IOTF reference systems are recommended to be used as international references [17]. However, national references appear to be more valid for the respective local population [17, 18].

To emphasize the impact of the reference population, we assessed the prevalence of overweight and obesity in a population-based registry of German and Austrian children and adolescents with T1D using WHO, IOTF, AGA, and KiGGS reference systems. We also compared rates of overweight and obesity over the period 2000–2013.

## 2. Methods

### 2.1. Data Collection and Study Population

The diabetes prospective follow-up (DPV) is an ongoing standardized registry for patients with diabetes [19, 20]. Anthropometric and laboratory data of patients with T1D were anonymized at all participating institutions and transmitted to the University of Ulm for central analysis. Quality of documentation was verified by reevaluation of inconsistent data and by DPV-benchmarking. The Ethics Committee at the University of Ulm and the local data protection officer approved data analysis.

Up to 2013, 356 specialized diabetes care centers in Germany (317 acute care clinics, 18 rehabilitation clinics) and Austria (21 acute care clinics) participated and contributed data for the present analysis. Data from subjects with T1D, aged between 2 and 18 years, were included. Children and adolescents with migration background were excluded to avoid the influence of ethnicity (*n* = 8,764). Migration background was defined as being born abroad or having a mother and/or father whose country of birth lies outside of Germany or Austria. Baseline characteristics are shown for the years 2000 (*n* = 9,461), 2004 (*n* = 13,014), 2009 (*n* = 16,011), and 2013 (*n* = 18,382) in Table 1.

The following diabetes-related parameters were analyzed: age at onset, diabetes duration, insulin/kg body weight and day, treatment by either conventional insulin treatment (CT, two injections per day) or intensified conventional insulin treatment and either by multiple daily insulin injections (MDI) or by continuous subcutaneous insulin (CSII, insulin pumps), and HbA1c (Table 1). HbA1c values were standardized to the Diabetes Control and Complications Trial (DCCT) reference range (20.7–42.6 mmol/mol) by the multiple of the mean (MOM) method [19].

T1D subjects were identified who participated repeatedly in this study. The numbers were *n* = 4892 for 2000 and 2004, 1582 for 2000 and 2009, and 406 for 2000 and 2013. Further, the prevalence of overweight and obesity in children with T1D was calculated considering three age groups: 2–<10 years, 10–<16 years, and 16–<18 years.

### 2.2. Weight Classification and Reference Systems

Weight status of German and Austrian T1D patients was defined separately according to four reference populations, provided by the World Health Organization (WHO) [13], the International Obesity Task Force (IOTF) [12], the guidelines of the German Obesity Association (AGA) [15], and the German Health Interview and Examination Survey for Children and Adolescents (KiGGS) [16].

### 2.3. Statistics

Statistical analyses were conducted using SAS, Version 9.4 (SAS Institute, Cary, NC). Results are given as mean ± SD or proportion (%).

BMI was calculated for all participants, who then were classified as overweight or obese depending on the WHO, AGA, and KiGGS cut-offs. IOTF provides cut-off points defined to pass through BMI of 25 and 30 kg/m^2^ depending on age at 18 years and sex. WHO system defines overweight as a BMI > 1 SD and obesity as a BMI > 2 SD from the mean of the WHO references population. AGA and KiGGS define overweight as a BMI above the 90th percentile of the reference population and obesity as a BMI above the 97th percentile [21].

Chi square tests were used for the comparison of overweight and obesity rates according to different references, age, and gender. Linear (continuous dependent variable) regression models were applied for the analysis of change in clinical characteristics over the years. Logistic regression models (binary dependent variable) were used to analyze the change of overweight/obesity rates or insulin therapy from 2000 to 2013. The regression models were adjusted for age and diabetes duration (both continuous variables) and sex. Year was included as continuous variable and *p* value for trend was calculated. Treatment center was entered as a random variable in these models in order to take between-center variation into account. The mean value during the first calendar year of each subject was used to calculate overweight and obesity prevalence in different age groups. A two-sided *p* value < 0.05 was considered to be statistically significant.

## 3. Results

### 3.1. Baseline Characteristics

Table 1 shows the clinical characteristics of study participants in the years 2000, 2004, 2009, and 2013. Slightly more males were recorded in the database. In recent years, T1D patients were on average three months older, heavier, and taller compared to 2000 (*p* < 0.0001, each). Insulin regimen in the respective years is given as well. In 2000, conventional treatment was used by 33.0% of the patients. This proportion decreased markedly to only 5.1% in 2013. The use of continuous subcutaneous insulin infusion increased from 3.5% in 2000 to 43.8% in 2013 (*p* < 0.0001). Most patients used multiple daily insulin injections, but the numbers decreased in recent years.

### 3.2. Overweight and Obesity Rate in T1D Subjects

**Figure 1:**
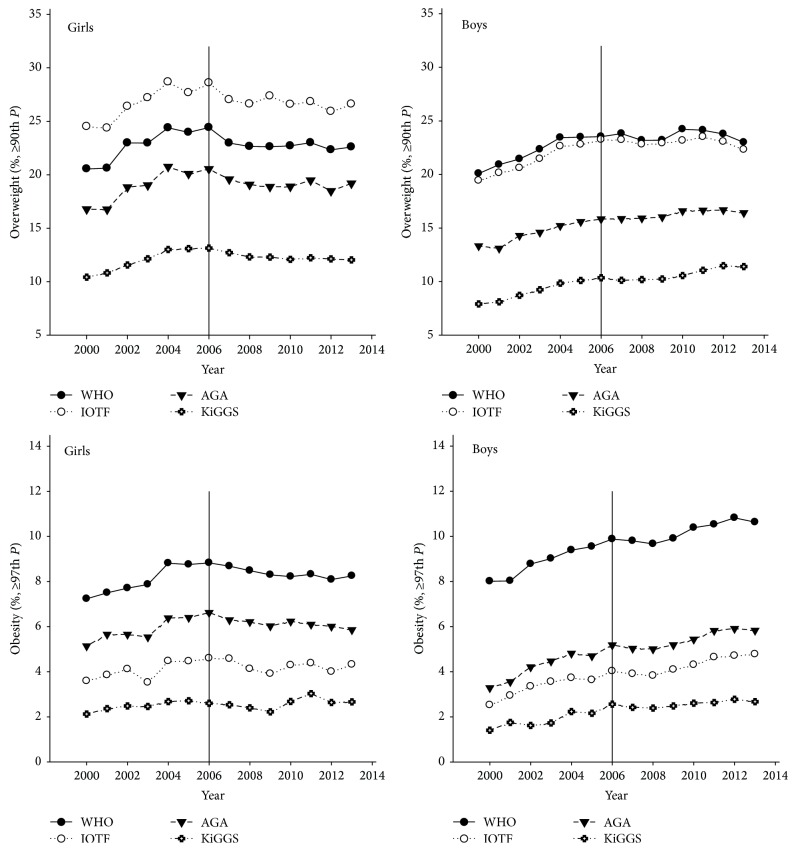
Prevalence (%) of overweight (including obesity) and obesity in girls and boys with T1D aged 2–18 years based on WHO, IOTF, AGA, and KiGGS reference population in the years 2000–2013, adjusted for age and diabetes duration. *P*: percentile. WHO: World Health Organization. IOTF: International Obesity Task Force. AGA: German working group on obesity in childhood and adolescents. KiGGS: German Health Interview and Examination Survey for Children and Adolescents.

The prevalence of overweight and obesity in T1D patients based on WHO, IOTF, AGA, and KiGGS reference and stratified for girls and boys is given in Figure 1. Assessed by the four reference populations, the prevalence differed significantly in each single year from 2000 to 2013 (all *p* < 0.0001). The difference between the references was up to 13% for overweight and up to 7% for obesity. Sole exception was the overweight rate in boys with a similar prevalence if evaluated by WHO or IOTF.

### 3.3. Overweight and Obesity Prevalence Related to Gender and Time Trend

Figure 1 shows that, in boys, the prevalence of overweight and obesity increased from 2000 to 2013 (*p* < 0.001, each reference). For girls, only the obesity rate based on KiGGS rose during the entire study period (*p* < 0.05).

In the years 2000–2006, both genders showed a significant increase in overweight and obesity rates according to all reference populations (*p* < 0.0001 each, obesity rate in girls based on KiGGS: *p* < 0.05).

In the years 2007–2013, in girls the overweight and obesity rates remained similar or even decreased slightly, as observed by the four references.

In the same time period (2007–2013), in boys the overweight rate increased further based on KiGGS only (*p* < 0.001) but remained stable according to WHO, IOTF, and AGA. On the contrary, the obesity rate in males increased significantly referring to WHO, IOTF, and AGA (*p* < 0.01) but remained stable based on KiGGS.

### 3.4. Overweight and Obesity Prevalence Related to Age and Gender

**Figure 2:**
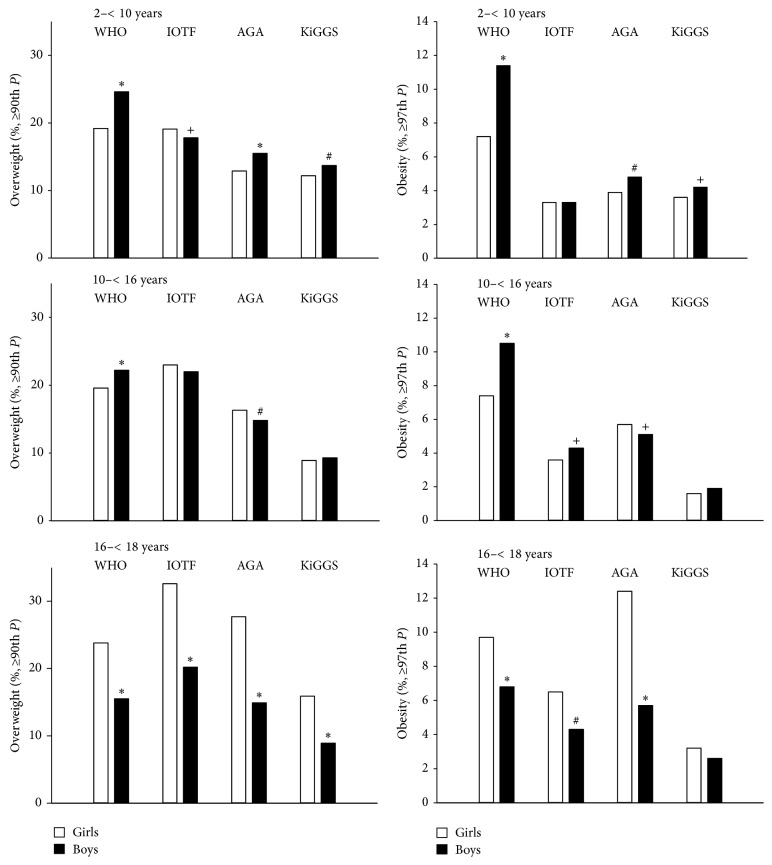
Prevalence (%) of overweight and obesity in girls (empty columns) and boys (grey columns) with T1D based on WHO, IOTF, AGA, and KiGGS reference population according to different age groups. *P*: percentile; ^*∗*^ *p* < 0.0001; ^#^ *p* < 0.001, ^+^ *p* < 0.05, boys versus girls.

The study population was divided into three age groups (2–<10, 10–<16, and 16–<18 years) (Figure 2). The comparison between genders revealed that in the oldest age group, independent of the reference used, always more girls were overweight than boys (*p* < 0.0001, each reference). Also, the prevalence was higher for obese girls, except for the KiGGS reference (*p* < 0.001 for WHO, IOTF, and AGA).

In the younger age groups, the rates for overweight or obesity were inconsistent according to the four references.

## 4. Discussion

The prevalence of overweight and obesity in German and Austrian T1D patients differed significantly across reference populations. Using IOTF or WHO reference data resulted in a high prevalence of overweight for girls and boys, whereas the comparison with more recent German references indicates lower rates. Pronounced gender differences were observed in children aged from 16 to 18 years with a higher overweight rate in girls according to all references.

Childhood and adolescent overweight and obesity are an epidemic and growing public health concern worldwide. As an easy measure in children and adolescents, obesity is determined by applying the body mass index with corresponding percentile charts for age and sex. There is a wide range of international and national systems to identify childhood obesity based on BMI, but no universally accepted system exists [18].

Growth charts are necessary to assess growth and weight gain of a child, since under- or overnutrition may be a health hazard. BMI as calculated by measurements of weight and height is neither time consuming nor costly and requires only a stadiometer and a regularly calibrated scale [22]. The use of the BMI as a measure for childhood obesity is common but has its disadvantages, since the BMI does not include the size of muscle tissue, bone density, the distribution of body fat, the bone tissue, and body water [23]. BMI is correlated with body fat but is not an accurate measure of fatness. The same BMI percentile does not represent the same percentage of body fatness at different ages, for boys and girls or for subjects of different ethnic origin.

The large differences in the prevalence rate for overweight or obesity, as calculated according to the four reference systems, have far-reaching consequences and raise many questions. How many children are classified as obese and are classified as being at risk for obesity-related diseases like hypertension, elevated triglycerides and total cholesterol, high LDL-cholesterol, or fatty liver? How many children should be screened for metabolic disorders? And should they be treated? Should children and adolescents be treated for obesity and/or the comorbidities [24]? Or should there be no treatment, because there is a possibility that weight normalizes with advancing age and growth in height? In contrast, several studies have shown that obesity during childhood is associated with persistence of obesity into adulthood [25]. Furthermore, it has been reported that children who are overweight/obese at young age are more likely to experience metabolic consequences in adulthood, even if the excess childhood weight is lost [26].

According to the four references, in the T1D subjects the overweight/obesity rates differ considerably. It is difficult to give a reliable assessment about the prevalence rates, without the knowledge on what reference basis. In clinical practice a physician would not be interested in the science that is necessary to establish appropriate thresholds and neither would be other health professionals or even governments/politicians, who should together fight the obesity epidemic. The complexity and discordance of childhood obesity apply to terminology, metrics, measures, reference values, and reference levels, but not at the last the statistics to bring it all together [27]. In a comprehensive and informative review Cole described “the development of growth references and growth charts” [28]. Usually a growth curve describes the growth (height, weight, or BMI) at a certain age, given as centiles, and displays the growth velocity over time by the slope of the curve. Over the last 200 years the growth charts have been more sophisticated, from simply giving height by weight, mean and SD centiles to taking into account growth in puberty [29, 30] and more suitable statistical methods [28].

Overweight and obesity in childhood and adolescence are a high risk for obesity in adulthood. Obesity, obesity-related diseases, for example, diabetes, and cardiovascular disease are disorders that rapidly increase in our modern society. However, long-term data and sufficient information are not available on the effect of BMI during lifetime on an individual's well-being, health, or disease, as well as costs on economic and healthcare systems. These data are essential to give valid recommendation for policies combating the obesity epidemic.

At present, a much debated topic is whether a growth chart should be applicable locally, nationally, or internationally. A reference chart originates from the anthropometry of a given population. The WHO reference was constructed from historical data of US children, and the objective was to develop a growth reference based on nonobese children [13]. IOTF reference was generated as an internationally acceptable definition for assessing children's weight from six large nationally representative cross-sectional growth studies [12]. National reference populations are often available but have been criticized, because they may be small in sample size, restricted to certain age ranges, or based on cross-sectional studies [31]. As shown in our study, the prevalence of overweight and obesity in the T1D subjects is quite different between AGA and KiGGS, although both use German reference populations. The KiGGS study was a recent investigation from 2003 to 2006 with the objective to compile a representative random sample with standardized data collection of the German paediatric population [16]. Data for AGA comprises measurements from 17 different cross-sectional surveys between the years 1985 and 1999 without standardized measurements. In recent decades, the average body weight has increased in children and adolescents worldwide, including German children. Therefore, in addition to the criteria that should be considered for the compilation of a reference chart, as outlined above, secular trends should be taken into account.

Recently, several studies investigated the applicability of the WHO reference compared to charts based on national populations [32, 33]. In our study compared to WHO reference, rates for overweight and obesity based on AGA and KiGGS were always lower, possibly due to the methods of data collection (AGA) and/or secular trends that affected KiGGS measurements. Both WHO growth charts and national charts were considered sufficient for the assessment of overweight/obesity rates, despite being not ideal [32, 33]. WHO reference data are established for clinical and epidemiological application [13], but defining paediatric obesity using national BMI reference data is just as widely recommended for clinical practice [17, 26]. Nonetheless, more research is demanded to identify the age-dependent BMI that is associated with adult pathologies.

Over the entire study period of 13 years, prevalence of overweight and obesity in girls and boys varied significantly. For boys the percentage of overweight/obesity was higher in 2013 compared to 2000, but in both years the frequency was similar for girls. For all children, a rise in the overweight/obesity prevalence was observed during the first years of data collection (2000–2006), but only in obese boys numbers rose further in recent years (2007–2013). Our study subjects covered children and adolescents with T1D and they usually receive nutrition counseling and take particular care with respect to their carbohydrate intake. Children with T1D eat fewer carbohydrates compared to healthy controls, but this lower carbohydrate intake may be compensated by increased fat consumption resulting in higher calorie intake and weight gain [34]. Possibly, the different prevalence rates may be explained by changes in diabetes therapy. Over the years few patients had CT and more subjects used insulin pumps. Higher doses of insulin were used by the patients, and the HbA1c level improved slightly in 2013 compared to 2000, due to the improved care for the patients, as has been suggested recently [35]. However, the changes in diabetes treatment were similar for boys and girls and therefore cannot explain solely the variability in overweight and obesity prevalence.

Yet, our findings confirm the well-established global increase in body weight documented in children during the last decades, and they are in accordance with some stabilizing trends seen recently in different countries [1, 2, 36]. Our results also confirm the finding that the stabilization is mainly observed in girls and the number of obese boys still increases, especially the number of severely obese boys [2, 37].

In our study, we observed a significantly higher rate of overweight and obesity in girls above 16 years of age. This finding is consistent with other studies, reporting a higher body weight in T1D girls compared to boys, primarily during puberty, although not all studies have shown a significant difference [38, 39]. In a recent study, risk factors for the increase in BMI were identified in T1D subjects. Particularly female gender, pubertal diabetes onset, intensive insulin therapy, and higher insulin dose were identified as risk factors for body weight gain [4].

The study is limited as the number of patients changed over the thirteen years and the smaller number in the first years may not be entirely representative for T1D children and adolescents in Germany. However, the increasing number is also due to increasing incidence of T1D. Further, in this multicenter approach, there may be some subjectivity and variability in data collection despite the standardized procedures.

The strength of our study is the high number of study participants, whose anthropometric and clinical parameters were measured under standardized conditions in specialized care centers and data were verified and analyzed in an independent research institute.

## 5. Conclusion

The prevalence of overweight and obesity in T1D children and adolescents differs significantly if it is assessed by four separate reference populations. This refers to individual classification of children's weight category, but also to scientific inference on the effect of age, gender, and time trend.

## Figures and Tables

**Table 1:** Clinical characteristics, HbA1c level, and insulin therapy of T1D study participants. Data are given for the years 2000, 2004, 2009, and 2013.

	Year of measurement				
	2000	2004	2009	2013	*p*
Number of participants	9,461	13,014	16,011	18,382	<0.001
Male (%)	51.8	52.2	52.9	52.5	<0.001
Age (years)	11.8 ± 3.8	12.0 ± 3.9	12.0 ± 3.9	12.2 ± 3.9	<0.0001
Age at T1D onset (years)	7.6 ± 3.8	7.6 ± 3.9	7.5 ± 4.0	7.5 ± 4.0	<0.0001
Diabetes duration (years)	4.2 ± 3.5	4.4 ± 3.6	4.5 ± 3.6	4.6 ± 3.7	<0.0001
Weight (kg)	46.7 ± 18.4	48.5 ± 19.4	48.7 ± 19.8	49.2 ± 19.6	<0.0001
Height (cm)	150.4 ± 21.7	151.2 ± 22.2	151.9 ± 22.3	152.7 ± 22.1	<0.0001
BMI (kg/m^2^)	19.6 ± 3.4	20.0 ± 3.8	20.0 ± 3.8	20.0 ± 3.9	<0.0001
HbA1c (mmol/mol)	64.2 ± 19.5	62.9 ± 17.8	63.4 ± 16.4	62.3 ± 16.3	<0.05
Insulin dose/weight (U/kg/d)	0.79 ± 0.29	0.84 ± 0.31	0.92 ± 0.34	0.95 ± 0.30	<0.0001
CT (%)	33.0	17.9	9.3	5.1	<0.0001
MDI (%)	63.5	70.8	58.3	51.1	<0.0001
CSII (%)	3.5	11.3	32.4	43.8	<0.0001

Unadjusted mean values ± SD, *p* values obtained from a regression model adjusted for age, gender, and diabetes duration, and *p* values for trend.

CT: conventional insulin treatment, MDI: multiple daily injections, and CSII: continuous subcutaneous insulin infusion.
